# Application of the Novel Two-Compartmental Model to Quantify Coronary Artery Calcium: A Pilot Study

**DOI:** 10.3390/jcm15051997

**Published:** 2026-03-05

**Authors:** Yu-Tai Shih, Zhe-Yu Lin, Jay Wu

**Affiliations:** 1 Department of Biomedical Imaging and Radiological Sciences, National Yang Ming Chiao Tung University, No. 155, Sec. 2, Linong Street, Taipei 112, Taiwan; n135724689@tzuchi.com (Y.-T.S.); a0905270576@gmail.com (Z.-Y.L.); 2 Department of Radiology, School of Medicine, Tzu Chi University, No. 701, Sec. 3, Zhongyang Rd., Hualien City 970, Taiwan; 3 Department of Medical Imaging, Hualien Tzu Chi Hospital, No. 707, Sec. 3, Zhongyang Rd., Hualien City 970, Taiwan

**Keywords:** coronary artery disease, coronary artery calcium score, computed tomography, two-compartment artery calcium score

## Abstract

**Background**: Cardiovascular disease (CVD) remains a major global health concern and the leading cause of mortality and disability. Early detection and prevention strategies rely heavily on evaluating coronary artery calcification, traditionally assessed using the coronary artery calcium score (CACS). However, CACS is limited by its dependence on strictly fixed tube voltage and slice thickness, sensitivity to changes in scanning parameters, and the need for an additional dedicated coronary calcium scan that increases radiation exposure. **Methods**: To address these challenges, we developed a novel two-compartment coronary artery calcium score system (TACS) for quantitative calcium assessment. TACS was established and validated using a QRM Thorax phantom scanned on a GE Revolution CT at 70–140 kVp. Volumetric calcium density (VCD) derived from TACS was compared with conventional CACS under varying slice thickness, pitch, and iterative reconstruction algorithms. Additionally, coronary artery calcium scans from 15 patients were retrospectively analyzed to assess correlations between TACS and CACS. **Results**: TACS demonstrated stable performance across tube voltages, with VCD errors ranging from 3.8% to −19.0% and maintained consistency under different slice thicknesses (23.9% to −2.3%) and reconstruction algorithms, showing near-zero residual percentages. Patient analyses revealed a strong correlation between TACS and CACS (r = 0.932). **Conclusions**: These findings suggest that TACS provides robust and reliable quantification of coronary calcium, supporting its potential use for opportunistic coronary artery disease screening, particularly in routine CT imaging. Further studies with larger cohorts are warranted to confirm its clinical applicability.

## 1. Introduction

Cardiovascular diseases (CVD) remain the primary cause of mortality worldwide [1,2]. Pharmacological prevention of CVD, informed by coronary artery calcium (CAC) quantification, is an established aspect of preventive medicine and is discussed extensively in guidelines from the American Heart Association [3] and the European Society of Cardiology [4]. CAC assessment has received a class IIa recommendation for individuals with borderline or intermediate 10-year risk for atherosclerotic CVD. These guidelines generally employ the coronary artery calcium score (CACS), commonly referred to as the Agatston score, which provides a semi-automated evaluation of calcification within the coronary arteries. For example, a CAC score of 0 may indicate postponing statin therapy and periodic reassessment, while a score of 100 or higher supports initiation of statin treatment [5,6,7].

Recently, several researchers have questioned the use of CAC measurement based on the Agatston score for various reasons. The fixed 130 kVp protocol restricts personalized adjustment of tube voltage according to patient body size [8,9]. Additionally, factors such as tube voltage, slice thickness, imaging modality, and reconstruction kernel can influence the accuracy of the Agatston score [9,10,11]. Furthermore, lowering the radiation dose remains important due to the potential cancer risk [11,12], and reliance on the traditional Agatston score may hinder the implementation of new CT technologies designed for improving CAC quantification [6,9].

To address these issues, multiple approaches have been suggested to improve CAC quantification by investigating the differences between scanning protocols. Some studies recommend employing the calcium mass score and the calcium volume score to reduce variability across scanning systems [10,13,14]. However, these alternatives are not widely utilized because of limited epidemiological data supporting their advantages in cardiovascular risk stratification over CACS. Several authors suggest different iterative reconstruction algorithms to increase the detectability of coronary artery calcium. Booij et al. [11] propose that calcium-aware iterative reconstruction methods may enhance detection and reduce variation in phantom scans. Willemink et al. highlight the importance of updating acquisition and reconstruction techniques to suit modern CT technology for accurate CAC quantification [15]. Bechtiger et al. [16] aim at the influence of different tube potentials on the calculated CACS. Photon-counting CT has shown increased spatial resolution and signal-to-noise ratio for coronary artery calcium detection [17,18,19]. Nevertheless, these aforementioned methods often require additional CAC scans to meet the parameters needed for Agatston scoring, leading to increased radiation exposure. Also, most current research continues to utilize the Agatston score protocols.

Our study proposes an alternative scoring system for measuring coronary artery calcification using a calcium mass-based approach, termed the two-compartment artery calcium score (TACS). In this study, the self-corrected two-compartmental model (TCM) was developed using regions representing the highest calcium concentration and phantom background as proxies for calcium and soft tissue. Previous research has described this model, indicating comparable results and potential application in abdominal CT for bone mineral density assessment [20,21,22].

The objectives of this study are: 1. to present a novel technique for quantifying coronary artery calcium using the two-compartment model (TCM); 2. to perform technical feasibility test in evaluating the diagnostic accuracy of TACS and CACS across varying scanning protocols using cardiac phantoms; and 3. to conduct a preliminary analysis of TACS in CAC scans, comparing its performance with CACS for coronary calcium quantification to assess the potential of clinical use.

## 2. Materials and Methods

In this study, two of the quantitative methods of coronary artery calcium were used:

The calcium score, also known as the Agatston score, is the reference standard for this study, as it remains the most widely accepted clinical metric, besides other scoring variations such as the calcium volume score and the calcium mass score [10]. It is calculated by the definition, according to Agatston et al., noted as below:

$$Agatston\;Score=\sum_{i}^{n}A_{i}\times w_{i}$$

In this formula, *n* means the number of calcified plaques whose area is above 1 mm^2^, A means the area of the calcium plaque, and w means the weighting factor of coronary calcium, listed below:

$$w=\left\{\begin{array}{c} 1,\;if\;130\;Housefield\;unit\;(HU)\leq I_{max}\\ 2,\;if\;200HU\leq I_{max}\\ 3,\;if\;300HU\leq I_{max}\\ 4,\;if\;400HU\leq I_{max} \end{array}\right.$$

*I_max_* means the maximal density of calcified plaque [14].

Our team has established the two-compartment model to calculate bone mineral density and has shown promising results for opportunistic examination by abdominal CT in the proximal femur [20] as well as the lumbar vertebra [21]. We developed a novel called TACS to estimate the mass of coronary artery calcium in the specific region of interest.

The CT number (CTN) of a mixture is defined as the ratio of attenuation coefficient to water, which can be calculated as below formula (k = 1000):

(1) $$CTN_{mix}=\left(\frac{{\bar{\mu }}_{mix}}{{\tilde{\mu }}_{water}}-1\right)\times k$$

The attenuation coefficient of the mixture can be estimated using the formula by the known attenuation coefficient *a* (${\tilde{\mu }}_{a}$) and *b* (${\tilde{\mu }}_{b}$) substance listed below,

(2) $${\bar{\mu }}_{mix}=\nu_{a}\times {\tilde{\mu }}_{a}+\left(1-\nu_{a}\right)\times {\tilde{\mu }}_{b}$$

We can estimate the volume of substance by a combination of Formulas (1) and (2):

(3) $$v_{a}=\frac{\left(\frac{CTN_{mix}}{1000}+1\right)-\left(\frac{CTN_{b}}{1000}+1\right)}{\left(\frac{CTN_{a}}{1000}+1\right)-\left(\frac{CTN_{b}}{1000}+1\right)}$$

By the definition of density, we can calculate the weight (*w*) of a substance by volume (*v*) and relative density (ρ) (density of a substance to density of mixture)

(4) $$w_{a}=\nu_{a}\times \frac{\rho_{a}}{\rho_{mix}}$$

We can further calculate the density of the mixture using the formula listed below. In this formula, *CTN_a_*, *CTN_b_* and *CTN_mix_* mean the CT number of substance a, b and mixture.

(5) $$\rho_{mix}=\frac{\rho_{b}\cdot CTN_{a}-\rho_{a}\cdot CTN_{b}+\left(\rho_{a}-\rho_{b}\right)\cdot CTN_{mix}}{CTN_{a}-CTN_{b}}$$

Therefore, we can calculate the fraction of calcium volume (CVF) by Formulas (3) and (5):

(6) $$CVF=\frac{\left(\frac{CTN_{voxel}}{1000}+1\right)-\left(\frac{CTN_{soft}}{1000}+1\right)}{\left(\frac{CTN_{ca}}{1000}+1\right)-\left(\frac{CTN_{soft}}{1000}+1\right)}$$

And

(7) $$\rho_{voxel}=\frac{\rho_{soft}\cdot CTN_{ca}-\rho_{ca}\cdot CTN_{soft}+\left(\rho_{ca}-\rho_{soft}\right)\cdot CTN_{voxel}}{CTN_{ca}-CTN_{soft}}$$

We define TACS as the total calcium mass in coronary arteries by calculating CVF and the density of the selected voxel. The unit of the TACS is gram or milligram.

(8) $$TACS=\sum_{i}^{n}CVF_{i}\times V\times \rho_{i}$$

In this formula, *n* means the number of lesions of calcium. *CVF* means the fraction of calcium. ρ means the density of the selected voxel, and *V* is equal to the volume of the selected voxel. By the definition of TACS, we assume that TACS does not influence the scanning parameters, including tube current, slice thickness, reconstruction algorithm or pitch ratio.

**Figure 1:**
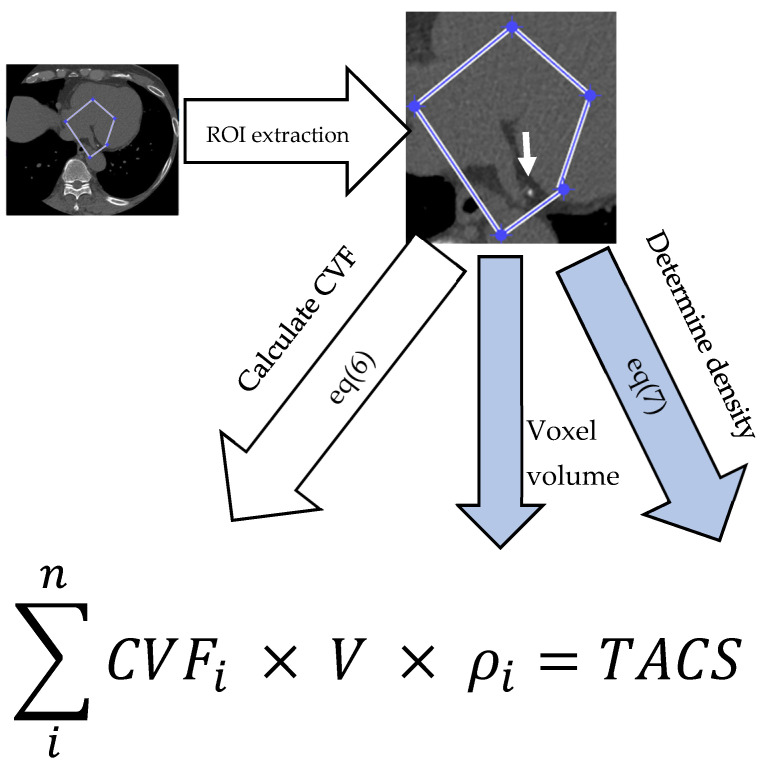
Graphic overview of the TACS acquisition and quantification process. TACS was calculated using custom-developed MATLAB code on selected CT slices. First, the Region of Interest (ROI) containing the calcification (white arrow) is extracted. For each voxel (i) within the ROI, three parameters are determined: (1) The Calcium Volume Fraction (CVF), calculated via Equation (6) using a threshold of 130 HU; (2) The Voxel Volume (V), derived from pixel area and slice thickness (2.5 mm); and (3) The Density of selected voxel (ρ), calculated via Equation (7). The final TACS represents the summation of the calcium mass across all voxels.

To build the algorithm to calculate TACS, A QRM anthropomorphic thorax phantom (QRM GmbH, Moehrendorf, Germany) was used to assess TACS characteristics. The phantom simulates different body sizes with small (300 × 200 mm), medium (350 × 250 mm), and large (400 × 300 mm) extension rings, and contains six calcification areas with hydroxyapatite concentrations of 200, 400, and 800 mg hydroxyapatite (HA)/cm^3^, corresponding to calcium density with 0.08, 0.16 and 0.32 g/cm^3^, each sized at 3 or 5 mm. Scans were performed using a GE Revolution CT scanner (GE Healthcare, Milwaukee, WI, USA). The protocol included varying tube voltages (70, 80, 120, 140 KVp), slice thicknesses (0.625, 1.25, 2.5 mm), ASIR levels (0%, 50%, 100%), and pitch ratios (0.508, 0.992, 1.375, 1.531). Volumetric calcium density (VCD) and TACS were calculated using home-made MATLAB code (Version R2024b; The MathWorks Inc., Natick, MA, USA). Manual region of interest (ROI) selection was performed to quantify the calcium volume based on the CVF, utilizing Equation (6), as well as the density of voxels in Equation (7) by density of calcium. In our experiment, we defined a similar threshold of coronary calcium in the Agatston score as 130 HU (Figure 1). A technical feasibility test was performed to evaluate the accuracy of the proposed TACS algorithm after the algorithms had been constructed. A total of 50 CT slices containing visible coronary calcifications were randomly selected from the 15 patients who underwent CAC CT scans at Hualien Tzu Chi Hospital, Hualien, Taiwan. These slices served as the validation dataset for the comparison between TACS and the standard Agatston score. Images were acquired in axial scanning mode using a tube voltage of 130 kV and a slice thickness of 2.5 mm, with automatic tube current modulation enabled. Standard CACS values were obtained using a CT workstation (AW workstation 3.2 ext. 3.4, GE Healthcare, Milwaukee, WI, USA), while TACS was computed via custom-developed MATLAB software (Version R2024b; The MathWorks Inc., Natick, MA, USA), as illustrated in Figure 1.

We perform a simple linear correlation between TACS and CACS among patients to compare their correlation. Data collection in this study received approval from the Institutional Review Board of Hualien Tzu Chi Hospital (IRB No. IRB112-240 B).

## 3. Results

### 3.1. CACS vs. TACS in Phantom

**Figure 2:**
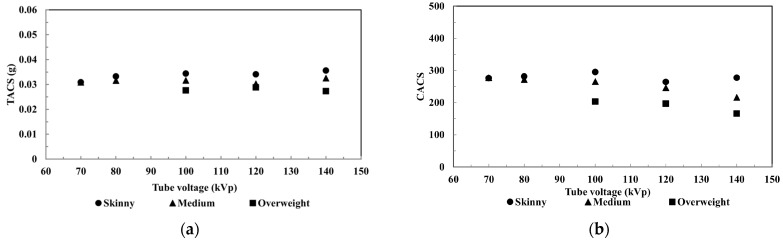
The (**a**) TACS and (**b**) CACS correspond to different phantom sizes and tube voltages.

**Figure 3:**
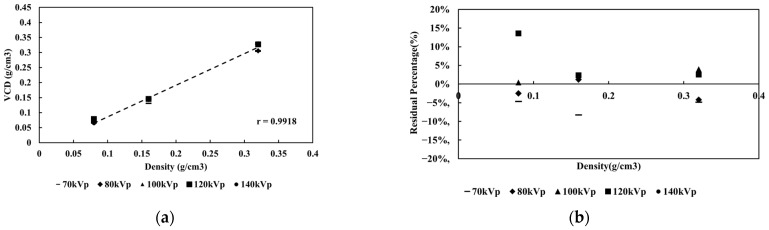
The relationship between the density of (**a**) CaHA and VCD and (**b**) RP% in different tube voltages.

Figure 2 and Figure 3 present TACS and CACS results for different QRM anthropomorphic phantom sizes and tube voltages. Consistent with prior research [10], CACS varies with scanning parameters: in medium and large phantoms, CAC scores decrease as tube voltage increases (277 at 70 kVp vs. 216 at 140 kVp), while small phantoms show no consistent trend (276–294 across voltages), possibly due to beam-hardening effects.

TACS remain stable regardless of tube voltage or fat ring differences, with calcium mass ranging from 30 mg to 36 mg in small and medium phantoms. For large phantoms, greater scatters are likely causing significant underestimation of TAC scores—up to 26.85% error compared to standard values—likely due to beam hardening. However, among large phantoms, TAC scores are similar across all tested voltages (0.028 g at 100 kVp, 0.029 g at 120 kVp, and 0.027 g at 140 kVp).

### 3.2. VCD Parameter Correlation

We utilized an anthropomorphic thoracic phantom, with the periphery of the phantom placed in a 300 × 200 fat ring to mimic a normal adult patient’s body habitus. Six different calcification points were analyzed in the images for their mean Hounsfield Units (HU), mean fraction of calcium volume (CVF), and Volume Calcium Density (VCD). The VCDs of the same HA concentrations were averaged and compared with the standard calcium density. The comparison results were evaluated based on the quality of linear regression fits and the calculated percentage error. Additionally, the relative percentage (RP%) was established to observe the normalized trend of VCD for calcification points with the same HA concentration.

#### 3.2.1. Tube Voltages

We estimated VCD in six calcium foci of a medium-sized phantom using various tube voltages (70, 80, 100, 120, and 140 kVp; Figure 2), finding a strong correlation (R^2^ > 0.99) across all settings. The RP% between VCD at different tube voltages ranged from –8% to +14%, indicating consistent results (Figure 2b).

#### 3.2.2. Slice Thickness

**Figure 4:**
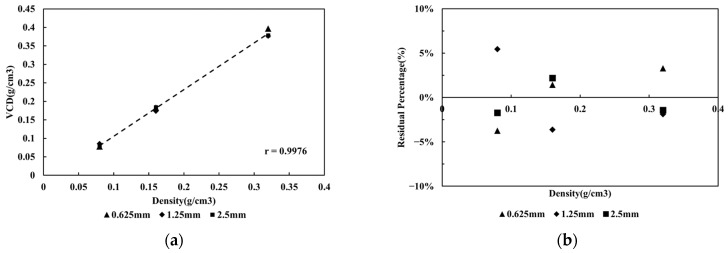
The relationship of calcium density and VCD among (**a**) different slice thickness and (**b**) in combination with the RP% of VCD in different slice thickness.

Slice thicknesses of 0.625, 1.25, and 2.5 mm were used for evaluation. The results showed that the linearity of the equations across the three slice thickness groups was consistent, with R^2^ values of up to 0.99. Figure 4 illustrates the relationship between slice thickness and TACS as well as RP%, as depicted in Figure 4a,b. The distribution of RP% ranged from −4% to 5%, indicating that using TACS, variations in slice thickness have minimal impact on VCD calculation compared to the mean VCD.

#### 3.2.3. Pitch Ratio

**Figure 5:**
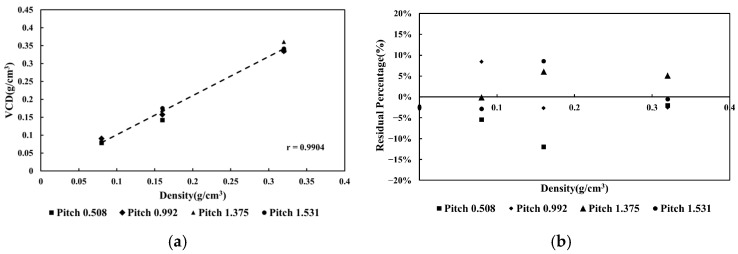
The relationship (**a**) of pitch ratio and VCD, and (**b**) the RP% of VCD among different pitch ratios.

The pitch ratios were determined as 0.508, 0.992, 1.375, and 1.531, respectively. For all four groups, the R^2^ values exceeded 0.99. Furthermore, after recombining the VCDs from the four distinct pitch settings and conducting a new linear regression analysis, Figure 5a shows that the linear relationship was largely preserved. Additionally, the minimum RP% observed was −12%, while the maximum was 9%, as shown in Figure 5b. The observed variation in RP% may be attributed to the standard deviation (or image noise) of CT numbers across different pitch ratios.

#### 3.2.4. Image Reconstruction Algorithm

**Figure 6:**
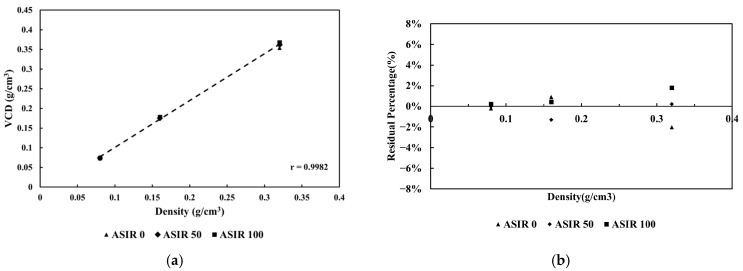
The relationship of the reconstruction algorithm and (**a**) VCD and (**b**) RP%.

The results indicated that varying reconstruction algorithms (ASIR 0%, ASIR 50%, ASIR 100%) did not result in significant differences in TACS quantification. The R^2^ values were 0.9976, 0.9995, and 0.9994, respectively, and the calculated VCDs closely matched the standard VCDs. The RP% deviation was limited to 2%, illustrating that the VCD results from each group were nearly equivalent to the mean VCD (Figure 6). These findings imply that changes in the four primary scanning parameters do not significantly affect the TACS. Moreover, the amount of standard calcium material was quantified accurately and reliably. Collectively, the phantom experiments confirmed the stability and reliability of TACS.

#### 3.2.5. Technical Feasibility Test

**Figure 7:**
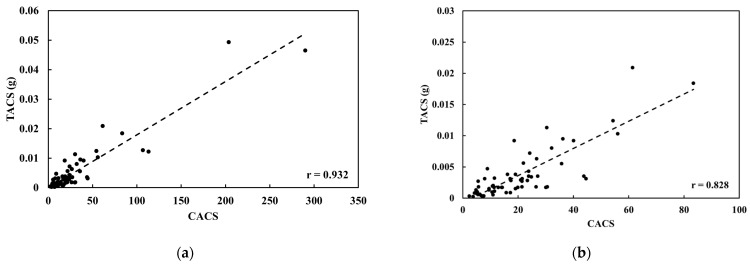
The comparison of CACS and TACS in 50 CAC CT slices (**a**) All slices and (**b**) CACS less than 100.

The preliminary comparison between CACS and TACS in clinical CAC CT in 50 slices among 15 patients is shown in Figure 7. There is a strong correlation (R^2^ = 0.868) between CACS and TACS within the 0 to 300 CAC score range. For small calcifications, the correlation coefficient was 0.686. These results indicate that TAC quantifies coronary calcification effectively and shows a substantial correlation between the two scoring systems.

## 4. Discussion

The present study signifies that TACS provides effective quantification of coronary artery calcification in CT images and addresses several limitations associated with the conventional CACS. Unlike CACS, which is highly dependent on scanning parameters such as tube voltage, slice thickness, and reconstruction algorithms, TACS exhibits stable performance across a wide range of imaging conditions. This stability enhances its potential for broader clinical application, including opportunistic screening using routine chest CT scans or even low-dose lung cancer screening, as previously demonstrated in a previous study using deep learning methods to obtain CACS [23].

Our phantom experiments confirmed that TACS maintains consistent results regardless of variations in tube voltage, slice thickness, pitch, and reconstruction algorithms. The accuracy, estimated by relative percentage error (RP%), remained low, indicating reliable volumetric calcium density (VCD) measurements. In contrast, CACS showed significant variability under different scanning protocols, which may limit its utility in diverse clinical settings.

Our study also aims to investigate the influence of different scanning parameters in TACS, which also has different characteristics from CACS. Bechtiger et al. investigated whether coronary artery calcification (CAC) scoring using 80 kVp and 70 kVp CT scans with tube voltage-adaptive scoring thresholds could achieve accurate cardiovascular disease risk stratification, compared to the clinical standard of 120 kVp. Using adjusted thresholds to calculate CAC scores could enable accurate risk stratification while reducing radiation doses [16]. Ki et al. recruited 20 participants for non-contrast chest CT (16 cm-coverage axial volume scan technique) for CACS, using the traditional Agatston method for CACS calculation. This showed the highest sensitivity observed in the 0.625 mm group for detecting calcified lesions. These studies show the variation in CACS in different scanning parameters, which is unlike TACS, showing similar results in most of the scanning parameters [24], which may suggest that diagnostic chest CT scans can be used for opportunistic CAD screening. The properties of TACS, which are shown in our study, enable us to use TACS to quantify coronary calcium. It will be our aim to study in the future.

In comparison to our study, Mergen et al. [9] evaluated the accuracy of CAC scores under varying tube voltages and monochromatic image reconstructions on the first-generation dual-source photon-counting detector CT. They found that using 65 kV with 90 kV and quantum iterative reconstruction (QIR) 3, as well as 70 kV with Sn100 kV and QIR 1, produced CAC scores consistent with those from traditional energy-integrating detector CT scans. For all phantoms simulating different patient body sizes, the percentage error was below 5%. The Mergen team concluded that the CAC score from dual-source PCD-CT is accurate across various tube voltages, offering the potential to significantly reduce radiation doses. In our study, our RP% is up to 14% in large body size, which probably resulted from the beam hardening effect, which will inevitably affect the result of TACS. Also, there is a fundamental difference between EID and PCD for signal processing to influence the signal-to-noise ratio, as well as different iterative reconstruction algorithms.

Despite these promising results, several limitations should be acknowledged. The clinical sample size in this study was relatively small, consistent with the nature of a pilot feasibility study. While the results demonstrate the technical applicability of the algorithm in vivo, they do not provide sufficient statistical power for comprehensive clinical validation. We acknowledge that manual selection of ROI is a variation in calcification selection in technical feasibility tests. However, according to our algorithm, the area of the ROI will not influence the result of TACS. Moreover, challenges remain in accurately identifying representative regions for calcium and soft tissue in clinical practice, which may affect the practicality and precision of the TACS method.

From a future perspective, the integration of artificial intelligence (AI), particularly deep learning algorithms, represents a rapidly evolving frontier in cardiac imaging. AI has demonstrated high accuracy in the automated segmentation of coronary calcium, significantly reducing the burden of manual annotation and inter-observer variability. The TACS method proposed in this study, while currently relying on manual ROI selection, is ideally suited for integration with AI-driven automated segmentation tools. A pipeline combining AI for rapid detection and localization of calcifications with TACS for precise, parameter-independent mass quantification could offer a powerful solution for large-scale, automated cardiovascular risk stratification. Furthermore, as AI facilitates opportunistic screening in non-ECG-gated chest CTs, the robustness of TACS against motion artifacts and acquisition variations becomes increasingly valuable.

The clinical utility of coronary artery calcium (CAC) scoring is expanding beyond traditional cardiovascular medicine. For instance, a recent meta-analysis by Cereda et al. [25] highlighted the need for calcium scoring in non-gated CTs. While the clinical prognostic value of incidental coronary calcification has been suggested in the recent literature, current assessments are limited by measurement heterogeneity across different scanning protocols. Our study addresses this technical gap. The proposed TACS method offers a parameter-independent quantification technique, theoretically minimizing the variability inherent in traditional scoring methods. Thus, the primary contribution of this work is providing a standardized technical solution that may facilitate more consistent data acquisition in future clinical investigations.

## 5. Conclusions

TACS offers a robust and parameter-independent approach for quantifying coronary artery calcium, potentially enabling more flexible and lower-radiation screening strategies. Future studies should focus on expanding clinical validation and refining the methodology for routine clinical implementation.

## Data Availability

The data presented in this study are available on request from the corresponding author. The data are not publicly available due to privacy.

## Abbreviations

The following abbreviations are used in this manuscript:

CAD	Coronary artery disease
CT	Computed tomography
CACS/CAC	Coronary artery calcium score/ coronary artery calcium
TACS	Two-compartment artery calcium score
TCM	Two-compartment model
CaHA	Calcium hydroxyapatite
CTN	CT Number
VCD	Volumetric volume density
RP	Relative percentage
